# Routes and rates of bacterial dispersal impact surface soil microbiome composition and functioning

**DOI:** 10.1038/s41396-022-01269-w

**Published:** 2022-07-01

**Authors:** Kendra E. Walters, Joia K. Capocchi, Michaeline B. N. Albright, Zhao Hao, Eoin L. Brodie, Jennifer B. H. Martiny

**Affiliations:** 1 Department of Ecology and Evolutionary Biology, University of California – Irvine, Irvine, CA USA; 2 Bioscience Division, Los Alamos National Laboratory, Los Alamos, NM USA; 3 Earth and Environmental Sciences, Lawrence Berkeley National Laboratory, 1 Cyclotron Rd, Berkeley, CA 94720 USA; 4 Department of Environmental Science, Policy, and Management, University of California, Berkeley, CA 94720 USA

**Keywords:** Community ecology, Microbial ecology, Bacteria, Fungal ecology, Soil microbiology

## Abstract

Recent evidence suggests that, similar to larger organisms, dispersal is a key driver of microbiome assembly; however, our understanding of the rates and taxonomic composition of microbial dispersal in natural environments is limited. Here, we characterized the rate and composition of bacteria dispersing into surface soil via three dispersal routes (from the air above the vegetation, from nearby vegetation and leaf litter near the soil surface, and from the bulk soil and litter below the top layer). We then quantified the impact of those routes on microbial community composition and functioning in the topmost litter layer. The bacterial dispersal rate onto the surface layer was low (7900 cells/cm^2^/day) relative to the abundance of the resident community. While bacteria dispersed through all three routes at the same rate, only dispersal from above and near the soil surface impacted microbiome composition, suggesting that the composition, not rate, of dispersal influenced community assembly. Dispersal also impacted microbiome functioning. When exposed to dispersal, leaf litter decomposed faster than when dispersal was excluded, although neither decomposition rate nor litter chemistry differed by route. Overall, we conclude that the dispersal routes transport distinct bacterial communities that differentially influence the composition of the surface soil microbiome.

## Introduction

Dispersal is the movement of individuals or propagules with potential consequences for gene flow [1]. This process has long been recognized as fundamental to the ecology and evolution of plant and animal communities [2–4]. More recently, evidence has accumulated that dispersal may also be important for microbiomes. Contrary to the long-standing assumption that microbial dispersal is so pervasive that it can be ignored [5], biogeographic patterns suggest that dispersal limitation influences the evolution and biogeographic distribution of microbial diversity [6–10]. Likewise, recent experiments that exclude immigration or artificially introduce cells demonstrate the potential for dispersal to alter microbiome composition and functioning [11–13]. While this evidence demonstrates the potential impacts of dispersal, we still have not measured the rates and taxonomic composition of dispersing bacteria or the impact of multiple dispersal routes on natural communities.

A dispersal route can be defined as the combination of the source community (e.g., soil or vegetation) and the physical vectors (e.g., rain or wind) that transfer individual cells. Two main attributes of dispersal routes—the rate at which individual bacteria move through them and the composition of those bacteria—are key to their influence on resident communities [14–16]. In laboratory microcosms, higher dispersal rates generally cause greater changes in resident microbiomes [17–19]. However, the impact of dispersal also depends on the taxonomic composition; a route transporting taxa that easily establish and grow in the resident community can have an outsized impact even under low dispersal rates [20, 21]. Thus, it is important to characterize both the rate and composition of a dispersal route to determine its impact.

Tracking the movement of microorganisms in the field, let alone characterizing dispersal rates, is a challenge. A handful of studies have followed subsets of microbial taxa, such as the accumulation of thermophilic endospores in sediments or the dispersal kernel for a single microbial taxon [22, 23]. Separately, dispersal routes have been inferred by the similarity between a focal community and potential source communities [16, 24, 25], although such inferences conflate the influence of dispersal and (unknown) environmental selection [26]. Other studies have experimentally blocked all dispersal or specific dispersal routes and then characterized changes in microbial composition. For instance, Kaneko and Kaneko [27] covered branches of beech trees to investigate the influence of dispersal on endophytic fungi, and Vannette and Fukami [28] caged flowers to restrict pollination to test for differential effects of animal pollinators on nectar-inhabiting microbial communities. Nonetheless, we still lack direct quantification of the rates and composition of microbial dispersal routes in natural ecosystems and tests of their individual impacts on microbiome composition and functioning.

**Fig. 1:**
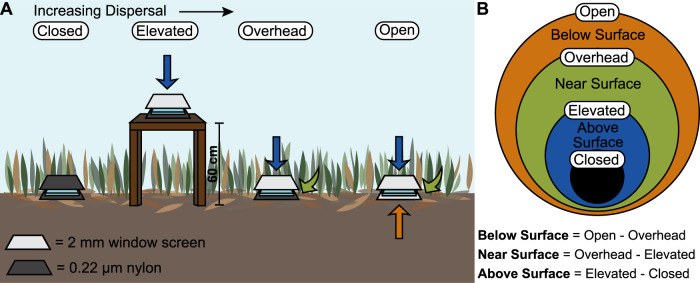
Overview of the dispersal experiment. **A** The four dispersal bag treatments contained one of two substrates, either glass slides or grass litter. The Closed treatment, a negative control, was placed on the ground and closed to all dispersal using a 0.22 µm nylon mesh bag. The Elevated treatment was placed on tables 60 cm above the surrounding vegetation in bags open to dispersal from above (2 mm window screen) but not below (0.22 µm nylon). The Overhead treatment was placed on the ground and open to dispersal from above (2 mm window screen) but not below (0.22 µm nylon). The Open treatment was placed on the ground and open to all dispersal (2 mm window screen). **B** Dispersal routes were isolated by comparing the treatments in a nested manner: contribution by the above surface route was inferred by the difference between the Elevated and Closed treatments; the near-surface route by the difference between the Overhead and Elevated treatments; and the below surface route by the difference between the Open and Overhead treatments.

To address these gaps, we characterized three potential bacterial dispersal routes and their impact on the surface soil microbiome of a southern Californian grassland. We asked three questions: (1) At what rate and by what routes are bacteria dispersing into the surface soil? (2) How do these dispersal routes influence microbiome composition in the soil surface? (3) Do the routes differentially influence microbiome functioning? We define the soil surface as the topmost layer of leaf litter, the most recently fallen addition to the soil. Below the surface is older, more senesced leaf litter and below that, the bulk (or mineral) soil. We aimed to compare three potential dispersal routes: *above surface, near surface*, and *below surface* (Fig. 1). We expected that the above surface dispersal route primarily includes dry and wet deposition from the regional pool of air down onto the soil surface. In contrast, the near surface route encompasses dispersal from live and standing senesced vegetation that is moved down and horizontally by wind, rain, or gravity onto the surface. Finally, the below surface route captures microorganisms that are transported by wind, rain, or capillary action up from the bulk soil and leaf litter [29] into the topmost litter layer. Previous work has identified bulk soil as an important dispersal source that contributes new taxa to leaf litter [30, 31], but its importance compared to other potential sources (e.g., air, leaf litter, vegetation) has not been studied.

We first characterized the abundance and taxonomic composition of bacteria immigrating into the soil surface by using sterile “traps”—glass microscope slides that allow little or no cell growth as they do not provide an energy source and limit moisture accumulation. To manipulate the exposure of the slides to the three routes, the slides were placed in different locations and enclosed in bags made of material that either allowed (2 mm window screen) or blocked (0.22 µm nylon) the immigration of bacteria, fungi, and larger organisms (Fig. 1). In a parallel experiment, we filled a second set of bags with freshly cut grass litter from the field site. This experiment allowed us to test whether the routes differentially altered the composition (bacteria and fungi) and functioning (decomposition rate and litter chemistry) of the resident surface microbiome.

## Materials and methods

### Field site

The experiment was conducted at the Loma Ridge Global Change Experiment in California, USA (33° 44′ N, 117° 42′ W, 365 m elevation) from April 14, 2018 to October 26, 2018. The site is a Mediterranean grassland (dry warm summers and cool wet winters), with 325 mm mean annual precipitation and 17 °C mean temperature, and is dominated by non-native grasses *Bromus madritensis* and *Avena* sp., non-native forbs *Hirschfeldia incana* and *Erodium* sp., and the native forb *Deinandra fasciculata* [32, 33].

### Dispersal slides and litterbags

We characterized dispersal through three routes: settling down from the air, moving horizontally from nearby surrounding vegetation (live vegetation and standing senesced grass), and transferring up from the bulk soil, which includes the surface litter layer. We measured dispersal onto two substrates: (1) sterile glass microscope slides to identify immigrating taxa; and (2) recently cut grass leaf litter collected on April 11, 2018, from the site. In total, the experiment encompassed eight dispersal treatments (four dispersal bag treatments × two substrates) and one death rate treatment. This design was replicated in seven experimental blocks (2 m by 2 m each) in an 11 m by 5 m field site.

The dispersal bags were made up of 0.22 µm nylon (Tisch, SPEC17970, North Bend, Ohio, USA) and/or 2 mm window screen (Phifer, Model # 300221, Tuscaloosa, Alabama, USA) depending on the treatment ( Fig. 1). Glass microscope slides (2.5 cm × 7.5 cm) were sterilized in 70% ethanol, dried, sealed into bags (5 cm × 10 cm), and autoclaved. Autoclaved litterbags (10 cm × 10 cm) were filled with green grass clipped into 2 cm segments and stored at 4 °C for up to three days before placement in the field. The bags were set out in the field on April 14, 2018, and were either staked to the ground or stapled to the field tables to secure in place. The samples on the soil surface were either placed on bulk (mineral) soil or the surface litter layer, depending on what was exposed. At each timepoint (May 23rd, June 13th, July 23rd, September 12th, and October 26th, 2018), we collected one bag from each treatment from each experimental block (9 treatments × 7 experimental blocks = 63 samples per collection). Glass slides were transferred to a sterile plastic bag with 2 mL of 1% phosphate-buffered glutaraldehyde (Pi-buffered GTA) and 220 µL of 0.1 M tetrasodium pyrophosphate and processed for community composition and bacterial abundance. Leaf litter samples were weighed, ground to homogenize, and processed for community composition (ITS and 16S rRNA gene amplicon sequencing), bacterial abundance, mass loss, and litter chemistry.

### Death rate slides

Bacterial death rate on the glass slides was measured so that immigration rate could be calculated using the bacterial abundance on the dispersal slides. To measure death rate, glass slides containing a known number of bacterial cells were placed into the field and sampled alongside the dispersal bags. Bacterial cells were extracted from grass litter from the field site by steeping the litter in 1 L of 0.9% saline solution overnight, stirred continuously. The litter was then filtered through cheese cloth, and the filtrate was aliquoted in 2 mL volumes, further concentrated and washed by pelleting the cells and resuspending into 100 µL sterile 0.9% saline solution. Each aliquot was then spread onto an ethanol-sterilized glass microscope slide and allowed to dry before being sealed into an autoclaved nylon (0.22 µm) bag that was closed to dispersal. The slides were kept at 4 °C overnight until being deployed in the field (on the soil surface). Timepoint 0 samples were suspended in 1% Pi-buffered GTA to preserve cell abundance for flow cytometry. One death rate sample was collected from each experimental block at each timepoint (described above) for a total of 35 samples (7 blocks × 5 timepoints). Additionally, a second set of death rate slides were deployed on both the soil and table surfaces on September 12, 2018, and sampled on September 19th, 26th, October 3rd, 10th, and 26th to calculate the death rate over a finer temporal scale and to capture the difference in death rates between the soil and table surfaces. All samples were processed for bacterial abundance and community composition (16S rRNA gene amplicon sequencing).

### Dispersal sources

To characterize potential sources of dispersal, we also collected air, soil, and environmental leaf litter samples from the field site (*N* = 3) at each timepoint (for a total of 15 samples each). To collect the air samples, we used the QuickTake 30 sampling pump with the BioStage single-stage impactor (SKC, Inc, Eighty Four, PA, USA) fitted with a sterile agar plate, collecting air from 4.5 m above ground for 30 min at 28.3 L/min flow rate. For the last timepoint, air samples were collected by directing airflow from a sterilized portable fan (O2COOL, model FD10101A) towards three vertical sterile agar plates for 30 min (although the different sampling methods may impact the community composition, these samples fall within the range of the air communities at other timepoints). All agar plates were kept at 4 °C for up to a week after collection. We removed a 4 cm × 4 cm area of the top 1 mm of agar using a sterile razor blade. To collect soil and litter, we pooled samples taken from the top layer of the bulk soil or the litter layer from three corners of each experimental block at each timepoint, repeating three times for the three samples per timepoint. Air and soil samples were processed for community composition (16S rRNA gene amplicon sequencing), and litter samples were processed for bacterial abundance and community composition (ITS and 16S rRNA gene amplicon sequencing).

### Bacterial abundance

At the time of sample collection, an aliquot of 0.1 g of ground and homogenized leaf litter was preserved in 5 mL of 1% Pi-buffered GTA and 550 µL of 0.1 M tetrasodium pyrophosphate. Glass slides were transferred to a sterile plastic bag with 2 mL of 1% Pi-buffered GTA and 220 µL of 0.1 M tetrasodium pyrophosphate. All samples were stored in the dark at 4 °C for up to two days before being sonicated for 30 min in the dark at 4 °C, filtered through a 4-µm-pore-size vacuum filter to remove large particulates, and stored in the dark at 4 °C for up to one day before being measured on the flow cytometer. To process samples on the flow cytometer, 2 µL of SYBR green (200×, Invitrogen Life Science Technologies, S756, Grand Island, NY, USA) was added to 400 µL of each sample filtrate, and samples were incubated in the dark at room temperature for 10 min. Samples were run for 30 s at 40 µL/min, using a SYBR-Green-H threshold value of 1500 and SSC-H threshold value of 1000. Gating parameters were used to count particles in the size of typical bacterial cells, optimized by Khalili et al. [34]. Statistical analyses were performed as described in the Supplementary Text.

### Amplicon sequencing

To characterize the bacterial community, we amplified the V4 – V5 region of the 16S rRNA gene using the 515F (GTGYCAGCMGCCGCGGTAA)—926R (CCGTCAATTCCTTTRAGTTT) primers, described in [35, 36]. To characterize the fungal community, we amplified the ITS2 region of the Internal Transcribed Spacer (ITS) using the ITS9F (GAACGCAGCRAAIIGYGA)—ITS4 (TCCTCCGCTTATTGATATGC) primer combination [37] (see Supplementary Text for further PCR and sequencing details).

Sequence data were processed in QIIME2 [38], version 2018.11 to identify exact sequence variants and assign taxonomy using the SILVA and UNITE databases for bacteria and fungi, respectively [39, 40] (Supplementary Text). We accounted for differences in sequencing depth among samples by rarefying to 1000 sequences (bacterial communities) or 3500 sequences (fungal communities) with 1000 resamplings. Notably, Bray–Curtis distance matrices of samples rarefied to 1000 versus 5000 sequences were highly correlated (Mantel tests: *R*^2^ > 0.99, *p* = 0.001). Further statistical details are provided in the Supplementary Text.

### Decomposition and litter chemistry

Decomposition, or mass loss, was measured as the percent decrease in dry weight. Dry weight was calculated by multiplying the wet weight of the leaf litter (both pre- and post-experiment) by the ratio of litter dry weight/wet weight. The ratio of dry weight/wet weight was calculated by drying a 1 g subset of the wet litter (taken at time of sample collection) overnight in a 60 °C oven until constant mass and dividing the dry weight by the initial wet weight. Ground and oven-dried litter collected at 2 months (on June 13, 2018) was analyzed for litter chemistry using attenuated total reflection Fourier transform infrared (ATR-FTIR) spectroscopy.

### Abiotic measurements

Precipitation, temperature, and wind speed data for the field site were collected from the weather station at the site maintained by the Center for Environmental Biology at the University of California—Irvine. To measure light intensity at the field site, we deployed Onset HOBO Pendant data loggers (UA-002-64, Onset Computer Corporation, Bourne, MA, USA) on the table and soil surfaces. Although these data were collected outside of our field experiment timeframe (February 15 to March 5, 2019), they allowed us to compare the relative light exposure of the soil and table surfaces. To assess temperature, we deployed iButton temperature sensors (Mouser Electronics, Mansfield, TX, USA) on the table and soil surfaces between September 12, 2018, and October 10, 2018. These data are described in the Supplementary Text.

## Results

### The rate and composition of bacteria dispersing into soil depends on the route

We destructively sampled the glass slides (*N* = 7) from four treatments (Closed, Elevated, Overhead, and Open) over six months, comparing cell abundances and taxonomic composition among the treatments. The four treatments allowed us to assess the contribution of the three dispersal routes to surface litter communities (Fig. 1). Closed samples were exposed to no dispersal. Elevated samples were only exposed to the above surface route, Overhead to the above and near surface routes, and Open to all three routes (above, near, and below surface). Where we can subtract data between treatments (i.e., for univariate data such as cell abundance and decomposition; subtractions done within replicate block), we report estimates by route. However, for data that cannot be subtracted between treatments (i.e., multivariate data including community composition and litter chemistry), we report the results by treatment—Closed, Elevated, Overhead, and Open—and infer route effects.

**Fig. 2:**
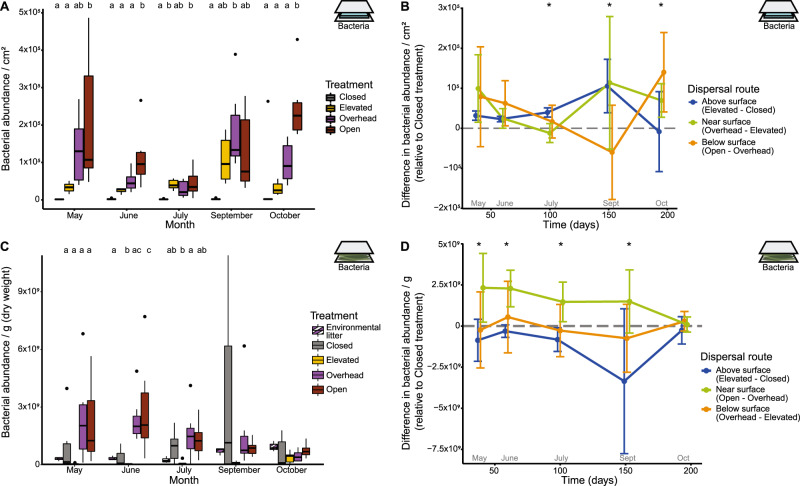
Bacterial abundance. **A** Bacterial abundance on the dispersal slides is significantly predicted by treatment (ANOVA: *p* < 0.0001, *R*^2^ = 0.30, F = 26.18), month (*p* < 0.0001, *R*^2^ = 0.11, *F* = 7.18), and the interaction between treatment and month (*p* < 0.001, *R*^2^ = 0.15, *F* = 3.20). Abundance differs by treatment in all months (*p* < 0.05). Letters indicate pairwise significance within each significant month (Tukey’s HSD). **C** Bacterial abundance in the litterbags is significantly predicted by both treatment (*p* = 0.00026, *R*^2^ = 0.12, *F* = 6.87) and the interaction between treatment and month (*p* = 0.015, *R*^2^ = 0.19, *F* = 2.06). Abundance differs by treatment for the first three months (*p* < 0.05) but not September (*p* = 0.12) or October (*p* = 0.53). Environmental litter is provided for context but was not included in statistical analyses. Abundance by dispersal route is calculated by the difference between treatments (Table S1) for **B** dispersal slides and **D** litterbags. Asterisks indicate that abundance differed by dispersal route (*post hoc* ANOVA: *p* < 0.05). Error bars represent 95% confidence intervals. Icons located in the top right of each panel indicate which experiment is being described: a blue slide for the dispersal slide experiment and green vegetation for the leaf litter experiment.

The average immigration rate of bacterial cells was similar through all three dispersal routes but varied over time in a route-dependent manner (Table S1; Fig. 2B). After accounting for cell death (3.34% of cells died daily; Fig. S1; see Supplementary Text), we calculated an average of 7900 bacterial cells/cm^2^/day immigrating onto the Open glass slides, equivalent to 0.47% of the average abundance in Open litter communities. Not all dispersal routes transferred cells onto the glass slides at every collection month. Immigration through the above surface route was significantly greater than zero during all months (one-sample *t*-test: *p* < 0.01) except October (*p* = 0.84), whereas immigration through the near surface route only occurred during May, June, and October (*p* < 0.05). Further, immigration through the below surface route was significantly greater than zero at the May, June, and October collections when the site received rainfall (*p* < 0.05; Fig. S2), suggesting that rain transferred cells from the soil and leaf litter up onto the slides at the surface.

**Fig. 3:**
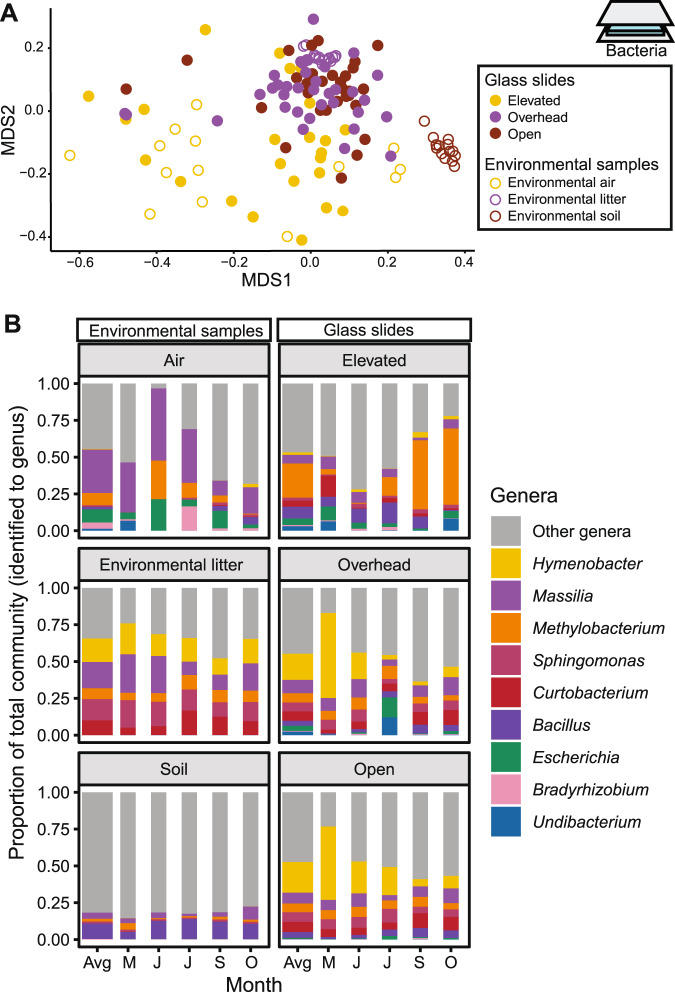
Community composition of dispersing bacteria. **A** Visualization (NMDS) of the composition of bacteria dispersing onto the glass slides and of bacteria from potential environmental sources. **B** The most abundant bacterial genera (relative abundance > 10% of community) in the environmental and glass slides samples, averaged (Avg) and by month (May, June, July, September, and October).

The taxonomic composition of dispersing bacteria also depended on the route (Table S2, Fig. 3), with large differences between the above and near surface routes. Bacterial composition on the Elevated slides appeared most similar to composition detected in environmental air samples (Fig. 3A). Although the two communities were still distinct (*post-hoc* test: *p* = 0.005), the centroid of the air samples was closer to the centroid of the Elevated samples (an average distance of 0.28) than that of the Overhead (0.36) or Open (0.40) samples. The Elevated communities had high proportions of the genera *Methylobacterium* and *Bacillus* (Fig. 3B), which are known to be viable in the atmosphere [41, 42]. Bacterial composition on the Overhead and Open slides differed from Elevated slides (*p* = 0.001) but not from one another (*p* = 0.35), and most closely resembled those in the nearby, environmental leaf litter (Fig. 3A). Their composition comprised a relatively even abundance of the genera *Hymenobacter*, *Massilia*, *Methylobacterium, Sphingomonas*, and *Curtobacterium* (Fig. 3B), taxa commonly observed in Loma Ridge leaf litter and known decomposers [33, 43–45]. Further, a SourceTracker analysis [46] estimated that 40% of the Overhead and Open communities could be traced to leaf litter, whereas fewer taxa were traced to soil (3.9%) and air (9.8%; Fig. S3).

The taxonomic composition of the glass slides also changed significantly over time and by the interaction between route and time (Table S2). In particular, Elevated samples showed higher variability throughout the course of the experiment (Fig. 3B; Fig. S4D), possibly reflecting the temporal variability of air communities [47].

### Dispersal routes differentially influence the leaf litter bacterial community

We investigated the influence of different dispersal routes on the surface litter microbiome. We constructed litterbags containing freshly cut grass from the field site to follow the successional shift from a phyllosphere to a decomposer community [48] in treatments parallel to the glass slide experiment (Closed, Elevated, Overhead, Open). The phyllosphere, which includes the leaf surface and the apoplast [49], contains diverse microbial communities that may contribute to the decomposer community as the leaf goes through senescence [30].

Bacterial abundance on the leaf litter was altered by dispersal in a route-specific manner (Table S3; Fig. 2D). In the absence of dispersal (Closed litterbags), abundance increased and then decreased over the course of succession (Fig. 2C). Dispersal from the above surface route did not increase bacterial abundance on top of this baseline pattern (one-sample t-test: *p* = 0.99; Fig. 2D). In contrast, dispersal from the near surface route resulted in significantly higher cell abundance (*p* < 0.0001), where the increase was greatest during the first month of the experiment (May) and diminished throughout the experiment. As a comparison, bacterial abundance in the surrounding litter increased throughout the experiment, although remained relatively low compared to the abundance within the litterbags (Fig. 2C). Moreover, additional dispersal from the below surface route did not further increase bacterial abundance over that of the near surface route (*p* = 0.61).

**Fig. 4:**
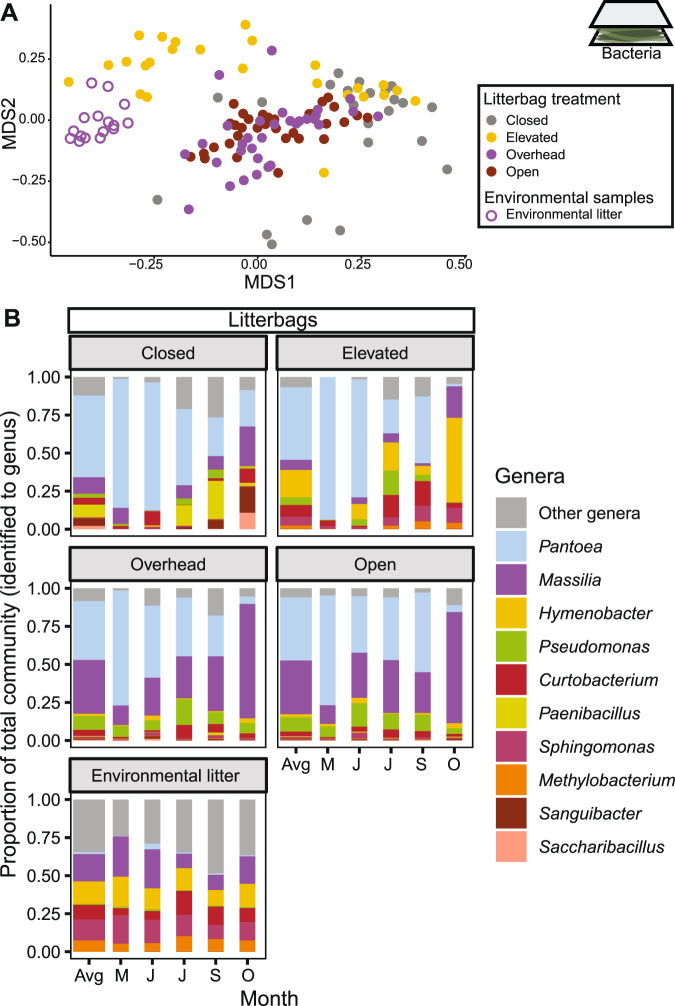
Community composition of litterbag bacteria. **A** Visualization (NMDS) of the composition of litterbag bacterial communities exposed to different dispersal routes including environmental (surrounding) litter as a comparison. **B** The most abundant bacterial genera (relative abundance > 9%) in the litterbag communities and the environmental litter, averaged (Avg) and by month (May, June, July, September, and October).

As with abundance, the composition of the litter microbiome was influenced by the dispersal route (Table S4A; Fig. 4A). Both the above and near surface routes influenced composition, whereas the below surface route did not (the Overhead and Open communities did not differ in community composition; *post hoc* comparison: *p* = 0.81). Bacterial community composition also changed over time, as expected during the succession of decomposing litter (Table S4A). However, dispersal also impacted this successional pattern, as indicated by a significant time-by-route interaction (Table S4A). Specifically, communities exposed to the near-surface route (Overhead and Open) resembled the composition of the surrounding environmental litter (a later stage of decomposition than the litterbags) after only a month (Fig. S5). In contrast, the Elevated treatment did not converge on the environmental litter composition until three months of decomposition, and the Closed litterbag communities remained dissimilar throughout the experiment.

The temporal dynamics of specific genera further illustrate the importance of dispersal route for microbiome succession on the leaf litter (Fig. 4B). *Pantoea*, a dominant grass phyllosphere member [50], was the most abundant bacterial taxa (81%) in the litter communities across all treatments after the first month. Over time, however, the communities that received dispersal through the near surface route (Open and Overhead) showed an increase (13% to 74%) in *Massilia*, an abundant genus in leaf litter at this site [11], whereas it remained at lower levels in other treatments from the beginning to the end of the experiment (21% and 26%). In contrast, the treatment exposed only to the above surface route (Elevated) showed an increase in *Hymenobacter* from undetectable to 55% at the end of the experiment. In the absence of all dispersal (Closed), *Saccharibacillus* and *Sanguibacter* became relatively more abundant (2% and 5%, respectively) than in the treatments open to dispersal (average of 0.1% and 0.3%, respectively). Further, dispersal through the near surface route (Overhead and Open) altered the overall alpha-diversity of the litter communities in ways consistent with later stages of litter decomposition [51], exhibiting higher Shannon diversity than Closed litter (*p* < 0.005; Fig. S4B). Overhead and Open treatments generally showed lower compositional variation (beta-diversity) than treatments not exposed to the near surface route (Closed and Elevated) (Fig. S4E; betadisper: *p* = 0.001).

### Dispersal via the above and near-surface routes accelerates initial litter decomposition

To test the influence of dispersal routes on community functioning, we tracked the rate of decomposition within the grass litterbags, comparing among treatments. Whereas all treatments plateaued in mass loss during the dry summer months, Elevated leaf litter exhibited a linear increase throughout the entire experiment. We hypothesize that this high rate of decomposition reflected increased photodegradation [52], rather than biotic decomposition, and removed this treatment from the decomposition and litter chemistry analyses (see “Discussion”).

**Fig. 5:**
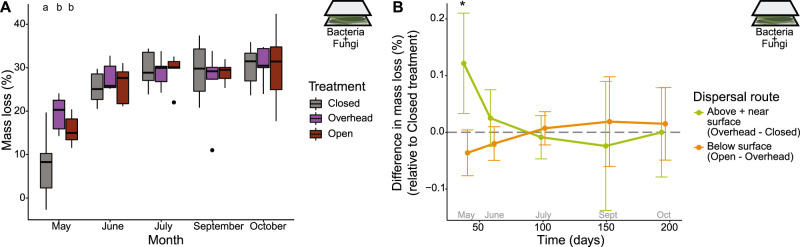
Mass loss within litterbags. **A** Treatment impacted mass loss in May (ANOVA: F = 10.02, *p* = 0.0012, *R*^2^ = 0.53) but no other months. Letters indicate pairwise significance within collection month (Tukey’s HSD). **B** Mass loss by dispersal route is calculated by the difference between treatments, grouping above and near surface routes together. May is the only month when mass loss differed significantly by dispersal route (Table S5), represented by an asterisk. Error bars represent 95% confidence intervals.

Among the remaining treatments, the litterbags lost on average 30.3% of their mass throughout the six-month experiment. Exposure to dispersal through the above and/or near surface routes increased mass loss during the first month of the experiment (one-sample t-test: *p* = 0.015; Table S5; Fig. 5B), during which Overhead and Open communities degraded leaf litter 2.5 times faster than Closed communities (Fig. 5A). The below surface route did not further increase mass loss above and beyond the above and near surface routes during the first month (*p* = 0.070). Since bacterial abundance in the litterbags was not correlated with decomposition rate (Spearman correlation: *p* = 0.16), dispersal from the above and/or near surface routes likely introduced specific taxa that were able to decompose dead plant litter more quickly. By the second month, however, all treatments degraded leaf litter at the same rate (Table S5; Fig. 5A) and, likewise, the chemical composition of the litter did not differ by treatment (Table S6; Fig. S6).

### Dispersal routes also influence fungal composition

Although our study focused on bacterial dispersal, fungi are also important decomposers in leaf litter at this research site [53]. We therefore also characterized fungal community composition in the grass-filled litterbags through ITS metabarcoding. Like the bacterial communities, dispersal via the above and near surface routes, but not below surface, influenced fungal community composition (PERMANOVA: *p* = 0.001; Table S4B; Fig. S7). Communities exposed to dispersal from above (Elevated, Overhead, and Open treatments) differed from Closed communities (*post-hoc* test: *p* = 0.001) and showed relatively higher proportions of the air-associated genera *Aureobasidium* (4.0%) and *Filobasidium* (4.1%) [54, 55] than the Closed treatment (1.3% and 0.7%, respectively). Likewise, the near surface exposed Overhead and Open communities differed from Elevated communities (*p* = 0.001) but not one another (*p* = 0.884), and appeared to have higher proportions of the decomposer taxon *Paraconiothyrium* (15.1%) than the Elevated or Closed communities (2.0%)] [56]. Further, like bacterial communities, increasing exposure to dispersal (Closed to Elevated to Overhead to Open) increased fungal alpha-diversity (Fig. S4C). The only pattern that differed from bacteria was that exposure to the vegetation route increased (rather than decreased) fungal beta-dispersion relative to the Closed and Elevated communities (PERMDISP: *p* = 0.001; Fig. S4F).

## Discussion

Here, we measured bacterial dispersal into the soil surface through three dispersal routes and characterized their effects on microbiome composition and functioning. The rates of bacterial dispersal through the routes were similar but remarkably low, despite the high abundance of bacteria in the surrounding potential sources, such as leaf litter and bulk soil, which both contain roughly 10^8^ cells g^−1^ dry weight [34]. At 7900 cells/cm^2^/day, the rate of incoming bacterial cells per area is high compared to estimates from seed and insect fall traps [57, 58]. However, this rate still makes up a small percentage (0.5%) of the community bacterial abundance in the Open litterbags. Given that leaf litter often forms a much deeper layer than that in the litterbags, this percentage is likely an overestimate. We therefore conclude that bacterial dispersal does not likely swamp the resident surface soil communities and lead to mass effects (*sensu* [59]). At the same time, despite its seemingly low rate, dispersal in this natural system altered the composition and functioning of the leaf litter microbiome. Previous mesocosm and modeling studies manipulated dispersal by adding pulses, or discrete additions, of bacterial cells to a resident community, making it difficult to directly compare to this continuous rate; however, in those studies, minimum pulses of 2.5–25% of total abundance were needed to impact community composition [17, 19, 60].

Two dispersal routes—above and near surface—transported a unique composition of bacteria into the soil surface, and both routes had unique impacts on microbiome composition. Microbial taxa dispersing through the above surface route most closely resembled taxa sampled directly from the air, whereas taxa from the near surface route resembled those found in the standing senesced grass and surface litter layer, likely transported by wind and rain onto the samples. In contrast, taxa dispersing through the below surface route were indistinguishable from the near surface route and did not resemble surrounding bulk soil. That is not to say that the bacterial immigration rate from the below surface route was negligible; the rate of dispersal from this route was, on average, equal to that of the other routes. Instead, the below surface route seemed to move cells from the litter to the topmost surface (the Open samples exposed below were placed on top of litter layer, if present). While bulk soil microorganisms may successfully colonize new litter in some systems [30, 31], bulk soil is perhaps less likely to be an important dispersal source in ecosystems with an annually persistent litter layer.

Exposure to the above and near surface routes changed the course of bacterial and fungal succession, shifting these communities towards the composition of the surrounding leaf litter, presumably reflecting a later stage of leaf litter succession. Given that the bacterial dispersal rates did not differ between the routes, we conclude that the taxonomic identities of the dispersing bacteria, rather than the overall dispersal rates, changed the resident community by outcompeting resident phyllosphere taxa. Alternatively, the dispersal limitation of individual taxa or the overall fungal dispersal rate could have contributed to the observed community changes, but these rates were not directly measured. Moreover, additional dispersal from the below surface route did not significantly affect leaf litter composition even though it increased the overall rate of bacterial immigration into the litter communities. Thus, we conclude that dispersal influences the resident community not through mass effects but rather through biotic interactions [21] between the species of bacteria and fungi present on the leaf litter and those immigrating in. Further, dispersal increased alpha-diversity and decreased bacterial beta-diversity among samples, supporting evidence from previous studies [19, 61] and metacommunity theory [59]. In contrast to bacteria, however, dispersal increased fungal beta-diversity. This discrepancy might be caused by the higher spatial and temporal heterogeneity of fungal versus bacterial communities in this system [33], perhaps leading to heightened priority effects [28, 62].

One caveat of our study is that the abiotic conditions on the tables and ground differed; the tables experienced lower temperatures and higher light exposure than the ground (Figure S8; Supplementary Text). Indeed, decomposition on the tables increased linearly over time, likely due to higher UV exposure. Differential selection may thus cause communities to appear distinct between the Elevated and Overhead treatments even if both treatments received the same immigrating taxa. Notably, however, the communities observed on the Elevated slides were highly similar to the communities identified directly from air samples, more so than those on the ground (Overhead and Open) (Fig. 3A), suggesting air as the primary dispersal source. For example, the most abundant genera on the Elevated glass slides (e.g., *Methylobacterium*, *Janthinobacterium*) were also abundant in air (Fig. 3B). In contrast, the alternative explanation - that cells are dispersing onto the Elevated slides from the near surface vegetation but then are being rapidly selected to look like the air community - seems less parsimonious and would necessitate differential death rates (since growth on the slides is highly limited or nonexistent). We thus conclude, despite not being able to entirely control the abiotic conditions, that the taxonomic composition of the above and near surface dispersal routes are distinct.

The differential impacts of the dispersal routes on microbiome composition also had functional consequences, at least during initial decomposition. Exposure to the above and/or near surface routes accelerated decomposition in the Overhead treatment during the first month of the experiment. Although we cannot tease apart the effect of these two routes (having removed the Elevated treatment in this analysis), we hypothesize that the increase in decomposition was due to dispersal through the immigration of well-adapted decomposers from the surrounding leaf litter onto the fresh green leaves. In support of this interpretation, dispersal through the near surface route alone increased bacterial abundance in the first three months of decomposition; past studies in this system demonstrate that higher decomposition rates lead to high bacterial abundance (rather than vice versa) [53, 63]. Although not directly measured, fungal immigration into the leaf litter may also have influenced decomposition; previous studies have identified both bacteria and fungi as driving decomposition at this field site [63, 64]. However, after two months, all communities— including those without dispersal—experienced similar levels of mass loss and similar litter chemical makeup. This attenuation shows support for previous findings that dispersal impacts are strongest during early succession [26].

While this study demonstrates the possibility of directly characterizing bacterial dispersal by different routes in an ecosystem, we also acknowledge limitations to the extent to which the results can be generalized even to other surface soils. Dispersal may be more important during environmental shifts or stressors because immigrating taxa adapted to the new conditions may outcompete resident taxa [20]. In particular, our experiment used freshly cut litter, which may be more susceptible to dispersal effects than litter in later stages of decomposition as the transition from green leaf to litter represents significant changes in the leaf environment (e.g., nutrient content, water availability). Further, the environmental context may impact dispersal routes and their influence on communities [21]. For instance, litter decomposition generally stops during the dry summer months at our site [64], and our results may have differed if the experiment was performed during the wet season instead. In fact, after an unusually heavy rainstorm for the season in October, the rate at which bacteria dispersed through the below surface route increased significantly, indicating that abiotic conditions may have a strong impact on dispersal rate by route. Along these lines, the dispersal routes in our study may also be specific to this leaf litter system. Our site comprises a thick leaf litter layer with minimal exposed bulk soil, perhaps explaining why dispersal through soil did not impact community assembly. A site with more exposed bulk soil would likely show different results, as we hypothesize that environmental context has a strong influence on dispersal. For example, a study conducted along the coast would likely observe influence from sea spray dispersal [65], yielding a different impact from dispersal through air on microbiomes.

## Conclusion

Our study suggests that dispersal impacts the surface soil microbiome in a route-dependent way, driven by differences in the taxonomic composition of the bacteria immigrating through different routes rather than by differences in dispersal rates. By adopting the experimental approaches demonstrated here to quantify dispersal routes in other systems, we can start to test how differences in the environment (e.g., precipitation, degree of wind) or ecosystem (e.g., plant community) impact microbial dispersal. For example, in this study, we observed seasonal variation in dispersal through the three routes, observing that the rates and taxonomic composition of dispersing bacteria vary over time. Thus, meteorological conditions may strongly influence microbial dispersal and, consequently, impact microbiome assembly. In this system, microbiome structure was mainly impacted by dispersal from surrounding vegetation and older leaf litter. If litter communities are primarily colonized, or “seeded,” by older decomposer communities, then disturbances such as fire that remove the litter layer “seed bank” may delay or prevent the assembly of decomposers in new leaf litter, the main source of soil nutrients [66].

## Supplementary information

Supplemental Material

## Data Availability

Sequence data generated from this work are available in the NCBI database under the BioProject number PRJNA841665. All other data and code are available on GitHub (https://github.com/kendraewalters/LomaRidgeDispersalRoutes).
