# Supplementary material for: Routes and rates of bacterial dispersal impact surface soil microbiome composition and functioning
Source: ISME J. 2022 Jul 1;16(10):2295–304. doi: 10.1038/s41396-022-01269-w (PMC9477824; doi:10.1038/s41396-022-01269-w)
Supplement: Supplementary file 1 — Supplemental Material [file 41396_2022_1269_MOESM1_ESM.docx]

**Supplementary Figures**

**Fig. S1**: **Results of the two death rate experiments. (A)** The abundance of bacteria cells remaining on the glass slides on the ground for the entirety of the field experiment (April – October; linear model: *R*^2^ = 0.81, *p* < 0.0001). Note that the regression lines are forced through a fixed y-intercept (mean abundance when time (days) is equal to 0), thus the *R*^2^ values are higher than if the regression did not have a fixed intercept. (**B**) Comparison bacterial cells remaining on glass slides on the ground (*R*^2^ = 0.81, *p* < 0.0001) and on tables (*R*^2^ = 0.80, *p* < 0.0001) for the last 1.5 months of the field experiment (September – October).

**Fig. S2**: **Weather data during the field experiment. (A)** Cumulative precipitation at the field site per day; (**B**) Average daily temperature at the field site; (C) Maximum daily wind speed at the field site. Vertical dashed lines represent sampling dates.

**Fig. S3: SourceTracker Results.** Proportion of bacterial community composition that was attributed to different source communities for Elevated (Kruskal-Wallis: *p* < 0.0001), Overhead (*p* < 0.0001), and Open (*p* < 0.0001) treatments. Letters indicate significant pairwise differences by dispersal route using Dunn’s post hoc test with a Bonferroni correction.

**Fig. S4**: **Alpha- and beta-diversity by dispersal route**. Shannon diversity differs by dispersal treatment for **(A)** bacterial communities on the glass slides (ANOVA: F = 5.22, *p* = 0.0073), **(B)** bacterial communities in the litterbags (F = 6.25, *p* = 0.00056), and **(C)** fungal communities in the litterbags (F = 62.88, *p* < 0.0001). Letters for panels A, B, and C indicate pairwise significance for each treatment (Tukey’s HSD). Beta-diversity (distance to group centroid) differs by dispersal route for **(D)** bacterial communities on the glass slides (betadisper: F = 8.2518, *p* = 0.002, *R*^2^ = 0.16), **(E)** bacterial communities in the litterbags (F = 10.533, *p* = 0.001, *R*^2^ = 0.21), and **(F)** fungal communities in the litterbags (F = 27.03, *p* = 0.020, *R*^2^ = 0.37). Letters indicate pairwise differences using a *post-hoc* test. Environmental samples are provided for context and are the same across all columns but rarefied independently. Icons located in the top of each column indicate which experiment is being described: a blue slide for the dispersal slide experiment and green vegetation for the leaf litter experiment.

**Fig. S5**: **Similarity to environmental litter.** Similarity to environmental litter was calculated as the similarity (1 – Bray-Curtis) between each sample and the centroid of the environmental litter samples each month. The distance to the environmental litter differs by treatment for all months (ANOVA: *p* < 0.05), except May (*p* = 0.16). Letters indicate pairwise significance within each significant month (Tukey’s HSD).

**Fig. S6. Litter chemistry composition by timepoint.** Visualization (PCA) of the composition of litterbag litter chemistry exposed to different dispersal routes, including environmental (surrounding) litter and initial litter (phyllosphere) as a comparison.

**Fig. S7**: **Fungal community composition of litterbag communities. (A)** Visualization (NMDS) of the composition of litterbag fungal communities exposed to different dispersal routes, including environmental (surrounding) litter as a comparison. **(B)** The most abundant bacterial genera (relative abundance > 4%) in the litterbag communities and environmental litter, averaged (Avg) and by month (May, June, July, September, and October).

**Figure S8: Light intensity and temperature on the table and ground surfaces.** (A) Light intensity (*p* < 0.0001) and (C) temperature (*p* = 0.0001) differed between the ground and table surfaces. (C) Light intensity and (D) temperature throughout the day for the ground and table surfaces.

**Supplementary Tables**

**Table S1**. ANOVA results for the effects of dispersal route, time (sampling timepoint), and dispersal route-by-time interaction on subtracted bacterial abundance on glass slides.

|  | Df | SumsOfSqs | F | *R*^2^ | *p* |
| --- | --- | --- | --- | --- | --- |
| Route | 2 | 9.35x10^9^ | 0.82 | 0.01 | 0.44 |
| Month | 4 | 4.43x10^10^ | 1.94 | 0.06 | 0.11 |
| Route-by-Time Interaction | 8 | 2.10x10^11^ | 4.60 | 0.28 | **0.0001** |
| Residuals | 85 | 4.85x10^11^ |  |  |  |

**Table S2**. PERMANOVA results for the effects of dispersal route, time (sampling timepoint), replicate block, and the interaction between route and time on bacterial community composition on glass slides.

|  | Df | SumsOfSqs | MeanSqs | Pseudo-F | *R*^2^ | *p* (perm) |
| --- | --- | --- | --- | --- | --- | --- |
| Route | 2 | 1.42 | 0.71 | 2.06 | 0.042 | **0.001** |
| Time | 4 | 2.39 | 0.60 | 1.72 | 0.071 | **0.001** |
| Block | 6 | 2.27 | 0.38 | 1.09 | 0.067 | 0.057 |
| Route-by-Time Interaction | 8 | 3.58 | 0.45 | 1.29 | 0.11 | **0.001** |
| Residuals | 1 | 23.90 | 0.35 |  |  |  |
| Total | 89 | 33.82 |  |  | 1 |  |

**Table S3**. ANOVA results for the effects of dispersal route, time (sampling timepoint), and dispersal route-by-time interaction on subtracted bacterial abundance in litterbags.

|  | Df | SumsOfSqs | F | *R*^2^ | *p* |
| --- | --- | --- | --- | --- | --- |
| Route | 2 | 1.23x10^20^ | 15.32 | 0.22 | **1.95x10^-6^** |
| Time | 4 | 3.33x10^19^ | 2.07 | 0.06 | 0.091 |
| Route-by-Time Interaction | 8 | 4.05x10^19^ | 1.26 | 0.07 | 0.27 |
| Residuals | 88 | 3.53x10^20^ |  |  |  |

**Table S4**. PERMANOVA results for the effects of dispersal route, time (sampling timepoint), replicate block, and the interaction between route and time on community composition in litterbags for A) bacteria and B) fungi.

**A) Bacteria**

|  | Df | SumsOfSqs | MeanSqs | Pseudo-F | *R*^2^ | *p* (perm) |
| --- | --- | --- | --- | --- | --- | --- |
| Route | 3 | 3.59 | 1.20 | 7.47 | 0.13 | **0.001** |
| Time | 4 | 3.09 | 0.77 | 4.82 | 0.11 | **0.001** |
| Block | 6 | 1.43 | 0.24 | 1.49 | 0.05 | **0.003** |
| Route-by-Time Interaction | 12 | 3.19 | 0.27 | 1.66 | 0.12 | **0.001** |
| Residuals | 92 | 14.73 | 0.16 |  |  |  |
| Total | 117 | 27.61 |  |  | 1 |  |

**B) Fungi**

|  | Df | SumsOfSqs | MeanSqs | Pseudo-F | *R*^2^ | *p* (perm) |
| --- | --- | --- | --- | --- | --- | --- |
| Route | 3 | 3.76 | 1.25 | 22.95 | 0.31 | **0.001** |
| Time | 4 | 0.83 | 0.21 | 3.79 | 0.069 | **0.001** |
| Block | 6 | 0.45 | 0.08 | 1.38 | 0.037 | **0.01** |
| Route-by-Time Interaction | 12 | 0.98 | 0.08 | 1.50 | 0.081 | **0.001** |
| Residuals | 111 | 6.06 | 0.05 |  |  |  |
| Total | 136 | 12.11 |  |  | 1 |  |

**Table S5**. ANOVA results for the effects of dispersal route, time (sampling timepoint), and dispersal route-by-time interaction on subtracted mass loss (decomposition rate) in litterbags for A) all timepoints, B) May, C) June, D) July, E) September, F) October.

**A) All timepoints**

|  | Df | SumsOfSqs | F | *R*^2^ | *p* |
| --- | --- | --- | --- | --- | --- |
| Route | 1 | 0.011 | 2.25 | 0.03 | 0.14 |
| Time | 4 | 0.020 | 1.03 | 0.05 | 0.40 |
| Route-by-Time Interaction | 4 | 0.089 | 4.64 | 0.23 | **0.003** |
| Residuals | 56 | 0.27 |  |  |  |

**B) May**

|  | Df | SumsOfSqs | F | *R*^2^ | *p* |
| --- | --- | --- | --- | --- | --- |
| Route | 1 | 0.087 | 15.73 | 0.57 | **0.002** |
| Residuals | 12 | 0.066 |  |  |  |

**C) June**

|  | Df | SumsOfSqs | F | *R*^2^ | *p* |
| --- | --- | --- | --- | --- | --- |
| Route | 1 | 0.0071 | 3.57 | 0.23 | 0.083 |
| Residuals | 12 | 0.024 |  |  |  |

**D) July**

|  | Df | SumsOfSqs | F | *R*^2^ | *p* |
| --- | --- | --- | --- | --- | --- |
| Route | 1 | 0.00088 | 0.65 | 0.05 | 0.44 |
| Residuals | 12 | 0.016 |  |  |  |

**E) September**

|  | Df | SumsOfSqs | F | *R*^2^ | *p* |
| --- | --- | --- | --- | --- | --- |
| Route | 1 | 0.0065 | 0.58 | 0.05 | 0.46 |
| Residuals | 12 | 0.13 |  |  |  |

**F) October**

|  | Df | SumsOfSqs | F | *R*^2^ | *p* |
| --- | --- | --- | --- | --- | --- |
| Route | 1 | 0.00057 | 0.17 | 0.02 | 0.69 |
| Residuals | 8 | 0.027 |  |  |  |

**Table S6**. PERMANOVA results for the effects of dispersal route and replicate block on leaf litter chemical makeup of the litterbags from June (2^nd^ collection month).

|  | Df | SumsOfSqs | MeanSqs | Pseudo-F | *R*^2^ | *p* (perm) |
| --- | --- | --- | --- | --- | --- | --- |
| Treatment | 2 | 2435.8 | 1217.89 | 1.57 | 0.13 | 0.15 |
| Block | 6 | 6951.5 | 1158.58 | 1.49 | 0.37 | 0.11 |
| Residuals | 12 | 9312.7 | 776.06 |  |  |  |
| Total | 20 | 18700 |  |  | 1 |  |

**Table S7**. Number of samples by month and treatment for the (A) bacterial composition from the dispersal slide samples, (B) bacterial composition from the litterbags samples, and (C) fungal composition from the litterbag samples.

**(A) Dispersal slides, 16S rRNA gene dataset**

|  | **Treatment** | | | | | | |
| --- | --- | --- | --- | --- | --- | --- | --- |
| **Month** | Elevated | Overhead | Open | Air | Leaf litter | Soil | Death slides |
| May | 5 | 7 | 6 | 3 | 3 | 3 | 6 |
| June | 6 | 7 | 6 | 1 | 3 | 3 | 6 |
| July | 5 | 7 | 6 | 1 | 4 | 3 | 6 |
| September | 4 | 7 | 6 | 5 | 3 | 2 | 6 |
| October | 4 | 7 | 7 | 4 | 3 | 3 | 6 |

**(B) Litterbag samples, 16S rRNA gene dataset**

|  | **Treatment** | | | | |
| --- | --- | --- | --- | --- | --- |
| **Month** | Closed | Elevated | Overhead | Open | Leaf litter |
| May | 5 | 1 | 6 | 7 | 3 |
| June | 4 | 5 | 7 | 6 | 3 |
| July | 5 | 5 | 7 | 7 | 4 |
| September | 4 | 7 | 6 | 7 | 5 |
| October | 5 | 7 | 6 | 6 | 3 |

**(C) Litterbag samples, ITS gene dataset**

|  | **Treatment** | | | | |
| --- | --- | --- | --- | --- | --- |
| **Month** | Closed | Elevated | Overhead | Open | Leaf litter |
| May | 5 | 6 | 7 | 3 | 3 |
| June | 5 | 6 | 5 | 3 | 3 |
| July | 7 | 4 | 6 | 7 | 4 |
| September | 4 | 3 | 7 | 4 | 2 |
| October | 4 | 3 | 5 | 2 | 2 |

**Supplementary Text**

**Supplemental Materials and Methods –**

***Amplicon Sequencing.*** Aliquots of 0.05 g of leaf litter, 0.1 g of soil, 250 µL from the glass slide solution taken immediately before vacuum filtering, and the agar scraped from the air samples were frozen at -70°C and stored until extraction. DNA was extracted following the ZymoBIOMICS Microprep DNA Extraction Kit protocol, with the following modifications: (1) for all samples, maximum centrifuge speed was 2808 g, instead of 3500 g, and centrifuge time was increased at that speed from 3 min to 4 min and from 5 min to 7 min; (2) for glass slide and air samples only, bead beating was reduced to 3 minutes, instead of 5 minutes, to avoid shearing the DNA in these low biomass samples; and (3) proteinase K was added to glass slide samples (with the exception of 33 randomly selected samples) to help uncrosslink proteins caused by the addition of the GTA.

To amplify the 16S rRNA gene, we used 1 µL of template DNA from the leaf litter and soil samples for the PCR. For air and glass slide samples, we used 5 µL of template DNA. For all samples, the remaining PCR reaction contained 12.5 µL of AccustartII PCR ToughMix (Quanta BioSciences, Inc, Beverly, MA, USA), 0.5 µL forward primer, 0.5 µL reverse primer, and H2O to reach a final volume of 25 µL. Following an initial denaturation step at 94°C for 3 min, the PCR was cycled 30 times at 94°C for 45 s, 55°C for 30 s, and 72°C for 60 s, with a final extension at 72°C for 10 min.

To amplify the ITS gene, we used 1 µL of template DNA from the leaf litter and soil samples for the PCR. For air and glass slide samples, we used 5 µL of template DNA. For all samples, the remaining PCR reaction contained 12.5 µL of AccustartII PCR ToughMix (Quanta BioSciences, Inc), 0.75 µL forward primer, 0.75 µL reverse primer, and H2O to reach a final volume of 25 µL. After an initial denaturing step at 94°C for 3 min, the PCR was cycled 35 times at 95°C for 45 s, 50°C for 60 s, and 72°C for 90 s, with a final extension at 72°C for 10 min.

To prepare libraries for sequencing, PCR products were pooled at different volumes based on amplification brightness on gel pictures: high (1 µL), medium (2 µL), low (3 µL), very low (5 µL), and none (8 µL). Glass slide, environmental litter, air, soil, and death rate samples were pooled together for one sequencing run. Litterbag samples and environmental litter samples were pooled together for a second sequencing run. We sequenced the environmental litter in both runs to control for differences in sequencing runs. After pooling, libraries were purified using SpeedBeads magnetic carboxylate-modified particles (GE Healthcare UK Limited, Buckinghamshire, United Kingdom). Purified libraries were sequenced in two paired-end Illumina MiSeq runs (2 x 250 bp) at the Genomics High Throughput Facility, UC Irvine, Irvine, CA, USA. Final sample sizes are provided in Table S7.

***Bioinformatic processing and analysis***. Sequence data were processed in QIIME2 [1], version 2018.11. For samples in the first sequencing run, the forward reads were trimmed to 6 – 279 base pairs of the 16S rRNA gene segment. For samples in the second sequencing run, the forward reads were trimmed to 6 – 300 base pairs. The reverse reads from both runs were discarded. We used DADA2 [2] to define exact sequence variants and assigned taxonomy using classify-sklearn with, for bacteria, the reference SILVA database (v. 138) at a 99% OTU level [3] and, for fungi, the UNITE (v. 7.2) database [4].

***Statistical analyses.*** We accounted for differences in sequencing depth by rarefying samples to 1000 sequences (bacterial communities) or 3500 sequences (fungal communities) with 1000 resamplings. To test whether a rarefaction depth of 1000 sequences was appropriate, we also rarefied the bacterial communities to 5000 sequences and compared the distance matrices of both rarefaction depths (Bray-Curtis) with a Spearman’s mantel test. For each sequencing run (glass slide samples and leaf litter samples), the two rarefaction depths were significantly correlated (*p* = 0.001) with an *R*^2^ of 0.99 and 0.997, respectively, indicating that the lower rarefaction depth yields similar results as the higher depth.

For each resampling, we calculated a Bray-Curtis dissimilarity matrix, taking the median similarity values of all 1000 matrices using the ‘vegan’ package in R [5, 6]. Using this median matrix, we performed a PERMANOVA and post-hoc test in PRIMER+ [7, 8] to test for differences in community composition by treatment and collection month as fixed factors and replicate block as a random factor. Bacterial composition on the glass slides was further linked to the potential dispersal sources using SourceTracker, a Bayesian approach that estimates the proportions of a community that come from possible sources [9]. To analyze alpha-diversity, we used the rounded rarefied dataset to calculate the Shannon index in R and a linear mixed model using the R packages ‘lme4’ and ‘lmerTest’ [10, 11] to test for differences among treatments (fixed factor) with replicate block as a random effect. Beta-diversity (Bray-Curtis metric) among treatments was analyzed using the betadisper() function in the ‘vegan’ package in R, examining distance to the group centroid. The alpha- and beta-diversity analyses were performed on the bacterial communities on the glass slides and the bacterial and fungal communities in the litterbags. Finally, to analyze similarity of litterbag samples to environmental (surrounding) litter over time, we calculated the centroid of the environmental litter samples within each collection month using PRIMER+ and created a similarity (1 – Bray-Curtis) matrix between the environmental litter centroids and the litterbag samples. We then used an ANOVA in R to test the effects of the litterbag treatments on the similarity between each litterbag sample and the environmental litter centroid at each collection month and Tukey’s HSD within each collection month.

To isolate the contribution of each dispersal route on univariate data (bacterial abundance, mass loss), we subtracted data between treatments within the same experimental block in a nested fashion (Fig. 1). To analyze the contribution of dispersal route and time on bacterial cell abundance or mass loss, we performed a Type III ANOVA using the ‘car’ package [12] and Tukey’s HSD in R for each subtracted dataset (bacterial abundance on glass slides; bacterial abundance in litterbags; mass loss). We also tested whether dispersal increased subtracted bacterial abundance or mass loss by performing a one-sample t-test that tested the difference from zero for each dispersal route overall and by route at each timepoint. As a complement, we analyzed the contribution of dispersal route, time, and their interaction on the non-subtracted bacterial abundance and mass loss data using a Type III ANOVA and Tukey’s HSD in R. To test the relationship between bacterial abundance in the litterbags and the decomposition rate, we performed a Spearman’s correlation on the two variables. To analyze litter chemistry, we calculated the Euclidean distance between samples of the baseline-corrected dataset (between 900-1800 nm) and used a PERMANOVA from the ‘vegan’ package in R to test for differences in chemistry among treatments and replicate block (fixed factors), using the FTIR spectral data. To compare the light levels and temperature experienced on the soil and table surfaces, we performed a two-sampled Welch’s t-test to test for a difference between the ground and table datasets.

**Supplemental Results –**

***Abiotic conditions.*** The light intensity ranged from 0 to 275 557 lux at the field site, with an average of 44 715 lux during daylight hours of 6:30 am – 15:30 pm (Fig. S8). Overall, light intensity was slightly higher on the table surfaces than the ground surfaces (Welch’s t-test: *p* < 0.0001, t = 25.62). At 1:00 pm, the tables experienced 45% greater light intensity than the ground surface, a difference of 43 000 lux. On the other hand, the ground surfaces experienced slightly higher temperatures than the table surfaces (*p* < 0.0001, t = 30.17). Temperature at the field site averaged 21.8°C, with a maximum temperature of 42°C on the table and 58°C on the ground (Fig. S8).

***Equilibrium between death and immigration rate.*** The cells observed on the glass slides are not just the product of immigration but the difference between immigration and death rate. Therefore, to estimate immigration rate, we first needed to estimate death rate on the glass slides. At the start of the experiment, we set out sample bags closed to dispersal that contained a glass slide with a known number of bacteria on it. The bacteria were a community derived from leaf litter from the field site to keep the taxonomic composition consistent with the rest of the experiment. More work is needed to determine how death rate may differ among different bacterial taxa. We measured the decrease in abundance over time at each time point to model death rate.

Overall, 3.34% of the community dies every day, or 21.2% per week (Fig. S1A). To calculate immigration rate, we used the average cell abundance on the Open dispersal samples and the estimated death rate. Because abundance on dispersal samples does not increase or decrease over time, we assume that equilibrium between death and immigration was established before the first time point. At equilibrium, the number of cells immigrating is equal to the death rate times the number of cells observed (mean over the five timepoints). Using that equation, we calculated 7,900 cells/cm^2^/day immigrating every day, or 55,000 cells/cm^2^/week.

These calculations assume that death rate is constant for every sample, whether samples were placed on the ground or the table surface. We tested whether this was true by measuring weekly death rate on the table and the ground from September – October. Overall, the death rates did not differ between ground and table. The weekly death rate on the table (28.05%) was slightly, but non-significantly, higher than the death rate on the ground (23.92%; ANCOVA, *p* = 0.090; Fig. S1B). Therefore, we can conclude that our assumptions are appropriate. However, there is still a chance we are underestimating dispersal from the above surface route. While the death rate over the entire experiment did not differ, the relative abundances between the ground and table samples did not show a consistent trend among timepoints. If we underestimated the death rate in the table samples, then we likewise underestimated the above surface dispersal rate. Last, the table surface is exposed to higher light intensities (Welch’s t-test: *p* < 0.0001, t = 25.62), which might result in a higher death rate but would not be picked up by our nylon death rate samples as the nylon blocks UV exposure.

**References**

1. Bolyen, E, Rideout, JR, Dillon, MR, Bokulich, NA, Abnet, CC, Al-Ghalith, GA, et al. Reproducible, interactive, scalable and extensible microbiome data science using QIIME 2. Nat. Biotechnol. 2019; 37: 852–857.

2. Callahan, BJ, McMurdie, PJ, Rosen, MJ, Han, AW, Johnson, AJA, Holmes, SP. DADA2: High-resolution sample inference from Illumina amplicon data. Nat. Methods 2016; 13: 581–583.

3. Quast, C, Pruesse, E, Yilmaz, P, Gerken, J, Schweer, T, Yarza, P, et al. The SILVA ribosomal RNA gene database project: improved data processing and web-based tools. Nucleic Acids Res. 2013; 41: D590–D596.

4. Nilsson, RH, Larsson, KH, Taylor, AFS, Bengtsson-Palme, J, Jeppesen, TS, Schigel, D, et al. The UNITE database for molecular identification of fungi: handling dark taxa and parallel taxonomic classifications. Nucleic Acids Res. 2019; 47: D259–D264.

5. Oksanen, J, Blanchet, FG, Friendly, M, Kindt, R, Legendre, P, McGlinn, D, et al. vegan: Community Ecology Package. 2019.

6. R Core Team. A language and environment for statistical computing. 2020. R Foundation for Statistical Computing, Vienna, Austria.

7. Anderson, MJ, Clarke, KR, Gorley, RN. PERMANOVA+ for PRIMER: Guide to Software and Statistical Methods. *Primer-E* . 2008. Plymouth, UK.

8. Clarke, KR, Gorley, RN. Primer v6: User Manual/Tutorial. 2006.

9. Knights, D, Kuczynski, J, Charlson, ES, Zaneveld, J, Mozer, MC, Collman, RG, et al. Bayesian community-wide culture-independent microbial source tracking. Nat. Methods 2011; 8: 761–763.

10. Bates, D, Mächler, M, Bolker, BM, Walker, SC. Fitting Linear Mixed-Effects Models Using lme4. J. Stat. Softw. 2015; 67: 1–48.

11. Kuznetsova, A, Brockhoff, PB, Christensen, RHB. lmerTest Package: Tests in Linear Mixed Effects Models. J. Stat. Softw. 2017; 82: 1–26.

12. Fox, J, Weisberg, S. An {R} Companion to Applied Regression, Second. 2011. Sage, Thousand Oaks, CA.
